# Advanced Therapies for Diabetic Kidney Disease: A Critical Review of Gene-, RNA-, and Cell-Based Strategies to Address Residual Risk

**DOI:** 10.3390/diseases14100383

**Published:** 2026-10-19

**Authors:** Kristina V. Kitaeva, Valeriya V. Solovyeva, Albert A. Rizvanov

**Affiliations:** 1 Institute of Fundamental Medicine and Biology, Kazan Federal University, 420008 Kazan, Russia; krvkitaeva@kpfu.ru (K.V.K.); vavsoloveva@kpfu.ru (V.V.S.); 2 Division of Medical and Biological Sciences, Tatarstan Academy of Sciences, 420111 Kazan, Russia

**Keywords:** diabetic kidney disease, diabetic nephropathy, type 1 diabetes, type 2 diabetes, residual renal risk, adeno-associated virus, RNA therapeutics, mesenchymal stromal cells, extracellular vesicles, kidney-targeted delivery

## Abstract

Background. Diabetic kidney disease (DKD) can progress despite contemporary kidney-protective treatment, motivating the development of additional mechanism-based interventions. This critical narrative review evaluates whether gene-, RNA-, and cell-based strategies are ready to address this residual risk and identifies the evidence required for clinical translation. Methods. A targeted literature verification and update was undertaken for the revision, with emphasis on primary intervention studies, contradictory findings, and the distinction between target validation and product efficacy. Mechanistic and model system background is considered in relation to therapeutic selection. Results. Adeno-associated viral vectors offer potentially durable expression but face species-dependent renal tropism, immunity, and limited control after administration. RNA therapeutics permit time-limited modulation, although productive renal delivery and compartment-specific effects remain unresolved. Mesenchymal stromal cells and extracellular vesicles provide broader paracrine activity, with challenges in potency definition, biodistribution, and manufacturing. Autologous renal cell products have entered clinical testing but require individualized production and invasive delivery. Most gene and RNA evidence is preclinical; cell therapy trials provide feasibility or exploratory efficacy signals rather than definitive kidney protection. Conclusions. Thus, at present, progress toward routine DKD use requires reproducible renal target engagement, validated manufacturing, and confirmation of clinical benefit in randomized trials.

## 1. Introduction

Diabetes affected an estimated 589 million adults aged 20–79 years worldwide in 2024 [1]. Chronic kidney disease (CKD) is common in both type 1 and type 2 diabetes [2,3,4], and diabetic kidney disease (DKD) contributes substantially to kidney failure and premature death. The American Diabetes Association (ADA) and Kidney Disease: Improving Global Outcomes (KDIGO) recommend assessment of urinary albumin-to-creatinine ratio (UACR) and estimated glomerular filtration rate (eGFR), together with layered kidney-protective care [5,6]. Here, DKD denotes CKD clinically attributed to diabetes; diabetic nephropathy (DN) denotes its characteristic histopathological lesions [7,8]. Diabetes with CKD does not automatically establish DKD: non-diabetic or mixed kidney disease must be considered when interpreting biomarkers and selecting participants for mechanism-specific trials.

Renin–angiotensin system (RAS) blockade, sodium–glucose cotransporter 2 (SGLT2) inhibitors, finerenone, and glucagon-like peptide-1 (GLP-1) receptor agonists have changed the clinical benchmark, especially in type 2 diabetes [9]. Advanced interventions therefore need to demonstrate additional benefit beyond appropriate background treatment. Their biological plausibility alone cannot justify greater treatment burden or less reversible risk.

The aim of this review is to critically compare adeno-associated virus (AAV)-mediated gene transfer, ribonucleic acid (RNA) therapeutics, mesenchymal stromal cell (MSC) and extracellular vesicle (EV) products, and autologous renal cell-based strategies as potential add-on treatments for progressive DKD. We ask which observations establish a therapeutic mechanism, which validate a deliverable product, and which support clinical benefit. The contribution is a cross-platform appraisal linking evidence maturity, renal compartment, delivery, manufacturing, patient selection, and trial endpoints. Pathobiology and experimental models are retained only where they inform these decisions; this is not a comprehensive account of diabetes care or a report of original experimental findings.

### Review Scope, Literature Selection, and Evidence Appraisal

The bibliography assembled for the initial manuscript formed the starting point for this critical narrative synthesis. Searches combined “diabetic kidney disease” or “diabetic nephropathy” with AAV/gene therapy; small interfering RNA (siRNA), antisense, messenger RNA (mRNA), or microRNA-21 (miR-21); and mesenchymal stromal cells, extracellular vesicles, or rilparencel. Exact title and named-trial searches checked pivotal results, discrepant findings, and overlapping cohorts. No lower publication date limit was imposed; foundational work was retained where needed to interpret current platforms, and only material available by the update date was considered. The verification used web discovery of indexed records rather than an exhaustive database export. For the MSC expansion, named ClinicalTrials.gov records were retrieved directly and cross-checked against publications; registry status, enrollment type, dose, route, and posted results were distinguished from publication findings.

English-language primary studies were selected when they directly informed a renal target, delivery platform, DKD intervention, or human safety/efficacy question. Guidelines provided the standard-care context. Non-DKD studies were used only for explicitly identified platform or translational lessons; unrelated studies and unverified efficacy claims were not used to establish DKD benefit. Reviews supported background context but did not substitute for primary reports when interpreting pivotal trials. Evidence was assessed qualitatively by study system, comparator, endpoint, follow-up, delivery, and relevance to treated human disease. Related trial cohorts were identified to avoid treating them as independent confirmation. This revision-stage process was targeted and non-exhaustive; it was not a registered systematic review, duplicate-screened search, or formal risk-of-bias assessment. Accordingly, no pooled effect or numerical evidence certainty grade is presented.

## 2. Pathobiology Linking Measurable Signals to Therapeutic Targets

### 2.1. Interacting Metabolic and Hemodynamic Stress

**Figure 1:**
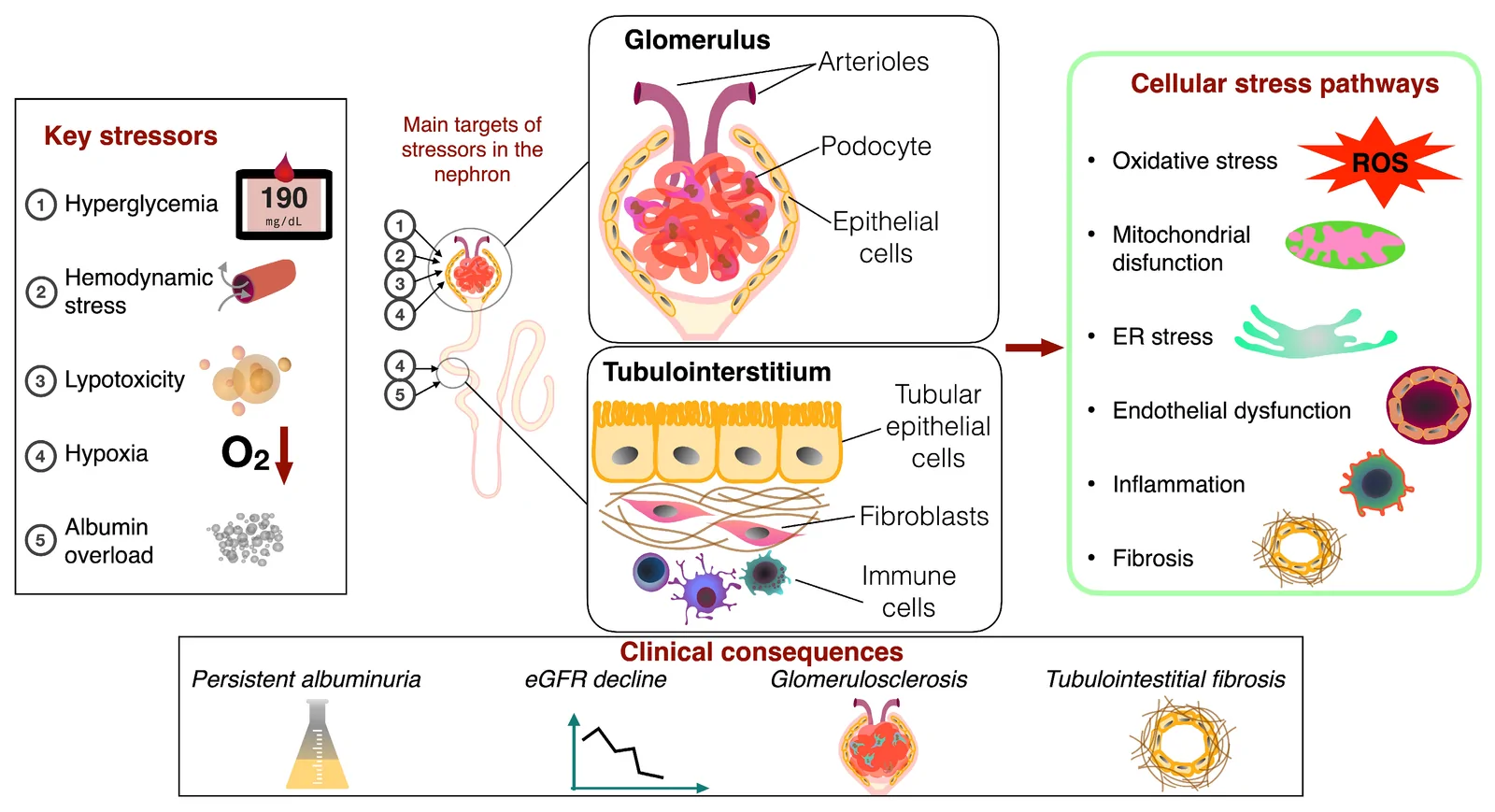
Simplified stress-centered pathogenesis of diabetic kidney disease (DKD). The diagram illustrates the interplay among major driving factors, their anatomical targets within the nephron, underlying cellular stress pathways, and ultimate clinical outcomes. Abbreviations: ROS, reactive oxygen species; ER, endoplasmic reticulum; eGFR, estimated glomerular filtration rate.

Hyperglycemia, altered lipid metabolism, and intraglomerular hypertension interact to produce reactive oxygen species (ROS), advanced glycation end products (AGEs), mitochondrial injury, and pro-fibrotic signaling [10,11,12,13]. These processes can persist in different renal compartments despite improvement in systemic metabolic control. Their relevance to advanced therapy is the distinction between an initiating stress and an established, potentially self-sustaining injury program. Figure 1 summarizes this mechanistic background; it does not imply that inhibition of any single pathway will reverse clinical DKD.

### 2.2. Cell-Specific Injury and Intercellular Crosstalk

Podocytes, glomerular endothelial cells, mesangial cells, and tubular epithelial cells exchange soluble mediators and EVs, linking filtration barrier injury to inflammation and fibrosis [12,14,15,16]. Podocyte depletion limits reversibility, whereas endothelial glycocalyx loss and impaired nitric oxide availability provide a rationale for barrier-protective approaches [17]. Consequently, reduced albuminuria may reflect altered permeability without replacement of lost cells.

Tubular epithelial cells face albumin overload, hypoxia, increased transport demand, and lipid stress. Their inflammatory signals recruit immune cells and activate neighboring stromal cells [12]. A treatment delivered mainly to proximal tubules therefore cannot be assumed to engage podocyte targets; conversely, a secreted protective protein may have effects beyond its producer cell.

Human single-cell and spatial profiling identifies distinct glomerular, tubular, immune, and fibrotic niches. A fibrotic microenvironment and, more recently, a B-cell-rich subgroup have been associated with progression [18]. These observations support biological stratification but do not establish that the identified cell states are causal, reversible, or predictive of response to a particular advanced therapy. Molecular signatures remain research tools until prospectively validated for treatment selection.

### 2.3. Oxidative Stress, Organelle Dysfunction, and Regulated Cell Fate

Mitochondrial dysfunction and impaired fatty acid oxidation connect metabolic stress to tubular injury and inflammatory signaling [11,13]. Endoplasmic reticulum (ER) stress and regulated cell death pathways, including apoptosis, pyroptosis, necroptosis, and ferroptosis, can amplify injury [19,20]. Their overlap argues against interpreting changes in one oxidative stress or cell death marker as evidence of organ-level rescue. The NLR family pyrin domain-containing 3 (NLRP3) inflammasome links cellular stress to interleukin-1β and interleukin-18 maturation [19,21]. Suppression of this pathway or activation of nuclear factor erythroid 2-related factor 2 (NRF2) can provide a pharmacodynamic readout, but neither is a validated surrogate for prevention of kidney failure. Such readouts should accompany compartment-specific functional assays and longer-term kidney outcomes.

### 2.4. Inflammation, Endothelial Dysfunction, and Fibrosis

Persistent inflammation, endothelial dysfunction, and capillary loss reinforce hypoxia and extracellular matrix accumulation [3,21]. Transforming growth factor-β (TGF-β) signaling interacts with Wnt/β-catenin and other profibrotic programs [22]. Because these pathways also participate in tissue homeostasis, prolonged or systemic inhibition requires a therapeutic window rather than an assumption that more suppression is better.

EVs can transmit either injury-associated or protective signals, depending on their source, cargo, and recipient cells [23,24]. Similarly, MSC products may influence several components of the inflammatory microenvironment, but this breadth does not itself demonstrate efficacy [25]. These distinctions motivate the product-specific appraisal in Section 5.

## 3. Contemporary Care and the Residual Therapeutic Gap

### 3.1. Contemporary Kidney-Protective Therapy

Contemporary kidney protection combines glycemic and blood pressure control with therapies supported by clinical outcomes. RAS blockade remains central when hypertension and albuminuria coexist; SGLT2 inhibitors reduce kidney events in eligible patients, including those with type 2 diabetes and CKD [26,27,28]. Finerenone provides additional kidney and cardiovascular protection in albuminuric type 2 diabetes [29,30,31]. The FLOW trial demonstrated kidney outcome benefit with semaglutide [32]. These findings establish the comparator for advanced-therapy trials rather than an efficacy estimate that can be transferred to a new platform.

In CONFIDENCE, empagliflozin plus finerenone reduced UACR more than either agent alone over 6 months [33]. This supports complementary activity but does not directly quantify additional long-term prevention of kidney failure. By the same reasoning, an advanced product that lowers albuminuria on top of standard care still requires evidence appropriate to its intended clinical claim.

### 3.2. Residual Risk Despite Contemporary Therapy

Despite major therapeutic advances, DKD continues to progress in a substantial proportion of patients. Persistent albuminuria and ongoing eGFR decline may occur despite optimized glycemic control, RAS blockade, SGLT2 inhibition, and other guideline-directed therapies, reflecting a clinically important residual renal risk [34]. This residual risk becomes particularly difficult to modify once podocyte depletion, tubulointerstitial injury, microvascular dysfunction, and fibrosis are established, because correction of upstream metabolic and hemodynamic abnormalities does not necessarily reverse pre-existing structural damage [35].

Residual risk includes irreversible nephron loss, persisting injury pathways, and incomplete implementation or tolerability of available treatment [18,34,35,36]. It is therefore necessary to distinguish biological treatment failure from undertreatment, non-adherence, intercurrent illness, or non-diabetic kidney disease before exposing a patient to an experimental product. Neither persistent UACR alone nor a single fall in eGFR proves that a specific advanced therapy is indicated.

### 3.3. Type 1 Diabetes and the Need for Additional Therapeutic Options

In type 1 diabetes, intensive glycemic management has durable kidney benefits, as shown by the Diabetes Control and Complications Trial and its Epidemiology of Diabetes Interventions and Complications follow-up [37]. RAS blockade is important when hypertension and albuminuria are present. Treatment effects established in type 2 diabetes require direct assessment in type 1 diabetes; for example, the ketoacidosis risk constrains SGLT2 inhibitor use.

FINE-ONE demonstrated a reduction in albuminuria with finerenone in adults with type 1 diabetes and CKD over 6 months [38]. The September 2026 United States prescribing information now includes reduction of UACR in this population, with an expected reduction in subsequent kidney risk [39]. This regulatory development should not be described as direct randomized evidence of fewer kidney failure events in type 1 diabetes. Jurisdiction-specific indications and the distinction between albuminuria and long-term outcomes remain relevant when defining background therapy.

Type 1 diabetes remains a relevant research population, but greater unmet need does not establish a more favorable benefit–risk balance for gene or cell therapy. Trials should distinguish diabetes types and account for disease duration, glycemic control, autoimmune context, and applicable background treatment instead of pooling patients without prespecified stratification.

## 4. Experimental Models for Target and Product Evaluation

### 4.1. In Vitro and Human-Derived Models

Two-dimensional human renal cell models are useful for target perturbation and early potency assays, but they represent selected injury mechanisms. Human kidney-2 (HK-2) proximal tubular cells have incomplete differentiation and transport characteristics [40,41]; conditionally immortalized human podocytes support barrier-related studies but do not reproduce an intact glomerulus [42]. Glomerular endothelial cultures can assess glycocalyx and permeability responses [43]. A result in one cell type should not be generalized to other renal compartments.

High-glucose experiments require osmotic controls and explicit reporting of glucose concentration, exposure duration, medium renewal, and cell phenotype [44]. Glucose depletion in control cultures can itself generate an apparent treatment contrast [45]. Rescue should therefore be confirmed with an orthogonal functional measure and a second relevant stress model, rather than inferred from a single viability or transcript assay.

Induced pluripotent stem cell-derived kidney organoids and microphysiological systems add human tissue architecture and, for chips, controlled flow [46,47,48]. Their immaturity, incomplete vasculature and immune context, and batch variability limit predictions of chronic adult disease. They can test human cell transduction, toxicity, and target engagement; they cannot establish systemic biodistribution, years-long safety, or prevention of kidney failure.

### 4.2. Animal Models and Translational Fit

Common rodent models reproduce selected DKD features more readily than progressive human kidney failure [49,50]. Streptozotocin (STZ) causes insulin deficiency but may directly injure tubules [51]. Akita and non-obese diabetic (NOD) mice avoid this chemical exposure while introducing genetic or autoimmune contexts, with strain-dependent progression [52,53]. These models are informative for mechanism but do not reproduce the full clinical history of treated type 1 diabetes.

The db/db and ob/ob models and Zucker diabetic fatty rats reproduce aspects of obesity, hyperglycemia, and renal injury; diet/STZ models provide another metabolic context [54,55]. Leptin pathway mutations, strain, sex, and study duration influence the phenotype [56]. Uninephrectomy or added hypertension can accelerate disease, including in renin-AAV db/db models [57,58,59], but the additional insult can change treatment responsiveness. Greater histological severity does not automatically confer greater predictive validity.

AAV tropism is species-, disease-, and route-dependent [60]. Strong mouse transduction does not establish productive delivery in adult human kidneys, and local delivery in a large animal may not predict performance after systemic administration. For MSCs, species compatibility, donor characteristics, pulmonary sequestration, and transient persistence affect interpretation [61,62]. Rodent doses, xenogeneic cell responses, and short follow-up cannot define a human dose or establish long-term safety.

For progression to clinical development, we propose evidence from more than one complementary disease or delivery system, human cell confirmation, randomized allocation and blinded outcome assessment where feasible, prespecified analysis, and independent replication. Studies should test treatment after injury is established and include clinically relevant background therapy when feasible. Both sexes, dose–response, target engagement, and adverse findings should be reported. These are proposed development criteria, not features verified in every study cited here; the absence of formal study-level risk-of-bias assessment limits conclusions about reproducibility.

## 5. Emerging Gene-, RNA-, and Cell-Based Strategies

### 5.1. Principles for Target and Product Selection

DKD is multifactorial, so a protective transgene or reparative cell product must be assessed as a treatment for a defined disease state rather than a correction of a single causal mutation. We distinguish three evidentiary steps: biological target validation; product validation, including delivery, activity, and safety; and clinical outcome validation. Human molecular associations alone satisfy none of these steps completely. Table 1 compares platform feasibility and proposed niches.

A candidate target should have human relevance and a measurable response in the intended compartment. The complete product—payload, promoter or formulation, dose, route, duration, and manufacturing process—must then be evaluated [63,64,65,66,67]. Biodistribution should distinguish vector or cargo detection from productive expression and biological activity [68,69]. A secreted protein can act beyond the transduced cell, but circulating exposure and effects outside the kidney must also be measured. Intracellular targets require direct access to the relevant cell population.

Isoform, signal peptide, processing, and secretion affect whether a construct produces the intended active protein; transcript abundance or an immunoblot band is insufficient evidence of functional exposure [66,70]. Non-native affinity or epitope tags require justification because they may affect localization, stability, or immunogenicity [70,71]. A tagged research construct cannot be assumed to have the same therapeutic properties as an untagged clinical candidate.

### 5.2. AAV-Based Gene Therapy: Therapeutic Concepts, Renal Targeting, and Translational Constraints

In db/db mice, AAV-mediated expression of thrombomodulin domain 1 (TMD1) improved albuminuria and renal histology, with changes in nuclear factor-κB (NF-κB)/NLRP3 and NRF2 signaling [72]. This provides pathway-level proof of concept. It does not establish incremental benefit on current background therapy, independent replication of a clinical-grade product, or a kidney failure benefit in humans.

Klotho overexpression, recombinant protein, and peptide studies support antioxidant, endothelial, podocyte, and antifibrotic effects in experimental DKD [73,74,75,76]. The convergence supports further target validation, but distinct molecular forms and delivery methods cannot be pooled as evidence for one AAV product. A development candidate still needs a defined active form, exposure range, renal compartment, and assessment of mineral metabolism effects. Human DKD efficacy has not been established.

Podocyte-directed vascular endothelial growth factor C (VEGFC) expression improved albuminuria, glomerular permeability, and endothelial glycocalyx preservation in experimental type 1 diabetes [77]. This is a compartment-focused proof of concept, not evidence of reversal of advanced fibrosis or replacement of podocytes. Reproduction in additional settings, control of local and systemic exposure, and vascular safety are required before clinical prioritization.

Cross-species capsid selection yielded AAV.k13 and AAV.k20 with improved proximal tubular transduction; renal delivery was demonstrated in additional large animal settings [60]. AAV.cc47 also improved proximal tubular transduction in mice and human kidney organoids [78]. These are delivery platform results, not DKD treatment trials. Context-dependent screening further shows that capsid rankings can change with species, disease, and route [79]. In human kidney organoids, AAV exposure was associated with inflammatory and profibrotic stress in a serotype-dependent manner [80], indicating that vector-only toxicity should be evaluated separately from the intended protective effect.

AAV durability is both a potential advantage and a safety constraint. Pre-existing antibodies, induced immune responses, restricted redosing, off-target expression, and the difficulty of stopping exposure complicate use in a chronic disease with effective alternatives [81]. A product should advance only after renal cell-specific activity is demonstrated at a clinically feasible dose and route. Risk-based surveillance should address persistent expression, renal and systemic toxicity, and delayed adverse events; rare integration-related risks require product-specific consideration rather than claims of either obligatory integration or zero genotoxic risk [69,82].

### 5.3. RNA-Based Therapeutic Approaches

Small interfering RNA (siRNA), antisense oligonucleotides (ASOs), microRNA (miRNA) inhibitors or mimics, and messenger RNA (mRNA) address different tasks: transcript suppression, network modulation, or transient protein expression [83,84]. Their activity is time-limited relative to durable AAV expression, but duration depends on chemistry and formulation; some effects persist well beyond plasma exposure. Dose adjustment or treatment cessation is therefore not an instantaneous molecular off-switch.

miR-21 illustrates why apparently convergent preclinical evidence requires scrutiny. Pharmacological antagonism improved albuminuria and structural or inflammatory outcomes in diabetic mouse studies [85,86]. In contrast, genetic miR-21 deficiency worsened albuminuria and podocyte loss in another diabetic model [87]. Genetic deletion and time-limited inhibition are not equivalent interventions, but the discrepancy indicates that compartment, timing, and degree of suppression matter. In the separate, non-diabetic setting of Alport syndrome, the randomized HERA trial of the anti-miR-21 agent lademirsen was stopped for futility [88]. This does not disprove efficacy in DKD; it shows why animal antifibrotic activity cannot substitute for renal target engagement and controlled human outcomes.

Experimental TGF-β1-directed antisense or siRNA approaches changed target expression or matrix-related readouts, and connective tissue growth factor (CTGF), also termed cellular communication network factor 2 (CCN2), directed ASOs reduced renal injury in diabetic models [89,90,91]. These findings support further investigation rather than a clinically validated antifibrotic strategy. The negative human anti-TGF-β1 antibody trial discussed in Section 6.1 cautions against equating pathway relevance with efficacy of any particular product.

Other regulatory RNA targets, including the miR-27a-3p/tissue inhibitor of metalloproteinases 3 (TIMP3) axis, remain supported mainly by experimental evidence [92,93]. For renal RNA delivery, tissue accumulation must be distinguished from uptake into the intended cells and endosomal release [94]. mRNA–lipid nanoparticle (LNP) systems additionally face strong hepatic distribution for many formulations [84]. Renal retention may increase exposure without improving productive delivery and can increase toxicity. Sequence-dependent off-target effects, innate immune activation, and carrier toxicity require separate assessment, including after repeat dosing.

Lumasiran reduces urinary oxalate in primary hyperoxaluria type 1 through hepatic gene silencing [95]. This validates a clinically useful RNA interference mechanism, but it does not validate delivery into diseased renal cells. For DKD, mRNA–LNP remains a development platform requiring direct renal pharmacology and safety data, rather than an established treatment category. Repeated administration also imposes manufacturing, adherence, and cumulative exposure burdens.

### 5.4. Mesenchymal Stromal Cells, Extracellular Vesicles, and Renal Cell Products

#### 5.4.1. Clinical MSC Studies in Diabetic Kidney Disease

MSCs are investigated primarily for paracrine immunomodulatory, endothelial, antiapoptotic, and antifibrotic effects, rather than durable replacement of renal cells. Clinical interpretation must remain product-specific: selected bone marrow precursors, CD362-selected stromal cells, umbilical cord MSCs, and autologous adipose-derived MSCs differ in manufacture and summarizes selected DKD studies, distinguishing actual enrollment and reported outcomes from planned recruitment and protocol-defined dosing. Cell counts describe exposure, not equivalent potency across products [25,96,97,98,99].

Packham et al. randomized 30 adults with type 2 diabetes and eGFR 20–50 mL/min/1.73 m^2^ to a single intravenous infusion of rexlemestrocel-L (150 or 300 million allogeneic bone marrow-derived mesenchymal precursor cells) or placebo, with 10 participants per group and 60-week follow-up [96]. Week 12 renal function trends were exploratory; the study was not powered to establish sustained kidney protection or an optimal dose. No treatment-related serious adverse events were identified, but this sample cannot exclude uncommon or delayed toxicity.

The Novel Stromal Cell Therapy for Diabetic Kidney Disease (NEPHSTROM) program tested allogeneic CD362-selected bone marrow MSCs (ORBCEL-M). The 80-million-cell cohort randomized 16 participants (12 active, 4 placebo), with an exploratory eGFR slope signal but no significant measured glomerular filtration rate (mGFR) benefit over 18 months [25]. The 2026 report describes the 160-million-cell cohort (13 participants; 10 active, 3 placebo), in which neither eGFR nor mGFR differed significantly between groups [97]. These are two cohorts of one trial, totaling 29 participants, not independent confirmations. All participants were men. The planned 240-million-cell cohort was not conducted; the registry records trial termination for product expiry and recruitment constraints [25,100].

Sabir et al. evaluated a single 30-million-cell infusion of allogeneic bone marrow MSCs in stage II–IV DKD [98]. They reported 91 participants in intention-to-treat (ITT) analyses and 78 in per-protocol analyses. Six-month isotope-GFR favored MSCs per protocol (*p* = 0.013), whereas the continuous 6 month GFR comparison in the published ITT was nonsignificant (*p* = 0.981). Allocation counts also differ between the narrative (41/50) and that table (40/51). Six deaths and seven losses to follow-up complicate interpretation. Registration occurred after study completion, and the registry lists 78 participants [98]. These reporting and attrition concerns, together with short follow-up, limit confidence in a durable treatment effect.

Alatta et al. reported direct renal artery delivery of autologous adipose-derived MSCs [99]. Two participants enrolled, but only one received cells: 0.25 million/kg per infusion, given at baseline and month 4 after a pandemic-related delay from the planned month 3 dose. No infusion-related or MSC-related serious adverse events or procedural complications were observed in that recipient. The reported pre/post eGFR slope change from −7.0 to −1.8 mL/min/1.73 m^2^/year is an uncontrolled observation, not evidence of a causal treatment effect. The registry lists the trial as terminated because of investigator relocation [101]. This experience supports technical feasibility only; it cannot establish comparative efficacy or general safety.

Registered umbilical cord MSC protocols explore repeated intravenous exposure. NCT04125329 plans 15 participants receiving approximately 1 million cells/kg at weeks 0, 4, and 8, with assessment through week 60 [102]. NCT04216849 plans a randomized, placebo-controlled study of 54 participants receiving 1.5 million cells/kg at weeks 0, 8, 16, 24, and 32, with assessment through week 48 [103]. At the 12 October 2026 check, both records had unknown status and no posted results. These are planned regimens, not observed efficacy findings. For weight-based regimens, total cell exposure depends on body weight; fixed and weight-normalized doses are not directly interchangeable.

Taken together, the human studies support continued product-specific investigation, but not a class-wide claim of nephron regeneration or prevention of kidney failure. Intravenous and renal-artery approaches have not been compared directly, and available studies do not establish the optimal source, dose, or dosing interval. Future trials should report manufacturing failures, actual cell exposure, prespecified handling of deaths and missing GFR measurements, and treatment-related as well as all-cause adverse events. Efficacy requires concurrent randomized controls, stable indicated background care, and sufficiently long follow-up for sustained kidney-function preservation. Repeated allogeneic dosing additionally requires immune monitoring; absence of a signal in a small single-dose cohort cannot establish repeat-dose safety [25,96,97,98,99].

#### 5.4.2. Engineered MSCs, Extracellular Vesicles, and Renal Cell Products

Human umbilical cord MSCs and engineered MSCs have reduced inflammatory or fibrotic readouts in diabetic rodents [104]. Such studies do not show durable renal cell replacement. Source, donor, expansion, cryopreservation, route, and species compatibility can change activity; a modified product needs direct comparison with its unmodified counterpart and a mechanism-linked potency assay. Payload expression alone does not establish improved therapeutic performance. Engineering may introduce additional genetic stability and immunogenicity requirements.

EVs remove the requirement to administer viable cells but do not automatically remove immunological or thrombotic risk. In an STZ diabetic immunodeficient mouse model, MSC-derived EVs improved injury and fibrosis measure [105]; the model does not establish efficacy in immunocompetent humans or isolate a universally active cargo. Minimal Information for Studies of Extracellular Vesicles 2023 (MISEV2023) supports characterization and attribution of activity, but it is a reporting framework, not regulatory approval or a complete product release specification [106]. EV identity, purity, dose normalization, residual soluble material, stability, and a relevant potency assay remain essential. Viability and senescence apply to producer or administered cells, not to acellular EVs themselves.

Rilparencel uses autologous cells obtained by kidney biopsy and delivered by intrarenal injection. The phase 2 study randomized 53 participants between dosing strategies rather than against an untreated or sham control [107]. Its exploratory within-patient eGFR findings cannot separate treatment effects from changes over time, background care, or regression to the mean. Biopsy and injection risks, individualized manufacturing, and the absence of definitive kidney failure evidence remain central limitations. These findings justify further controlled testing; they do not establish renal regeneration.

### 5.5. Translational Requirements

The relative advantages in Table 2 are engineering and clinical development considerations, not demonstrated differences in patient benefit. AAV may reduce dosing frequency but is difficult to withdraw; RNA permits repeat-dose adjustment but requires a delivery solution; allogeneic MSCs may support banked manufacture; EV production requires scalable purification and potency control; autologous renal cell products require a patient-specific production chain. No head-to-head DKD trial establishes superiority or cost effectiveness among these platforms.

Manufacturing is part of the biological intervention. AAV programs need control of identity, genome integrity, potency, impurities, and lot consistency; RNA products need sequence and integrity testing together with formulation and delivery-linked potency. Cell products require identity, purity, sterility, viability, genetic stability where relevant, and a mechanism-related functional assay; freeze–thaw handling must preserve the intended activity. EV products need reproducible composition, impurity control, and activity per justified dose unit. Changes in scale, culture, purification, or formulation require comparability data rather than an assumption that products remain interchangeable [110].

**Figure 2:**
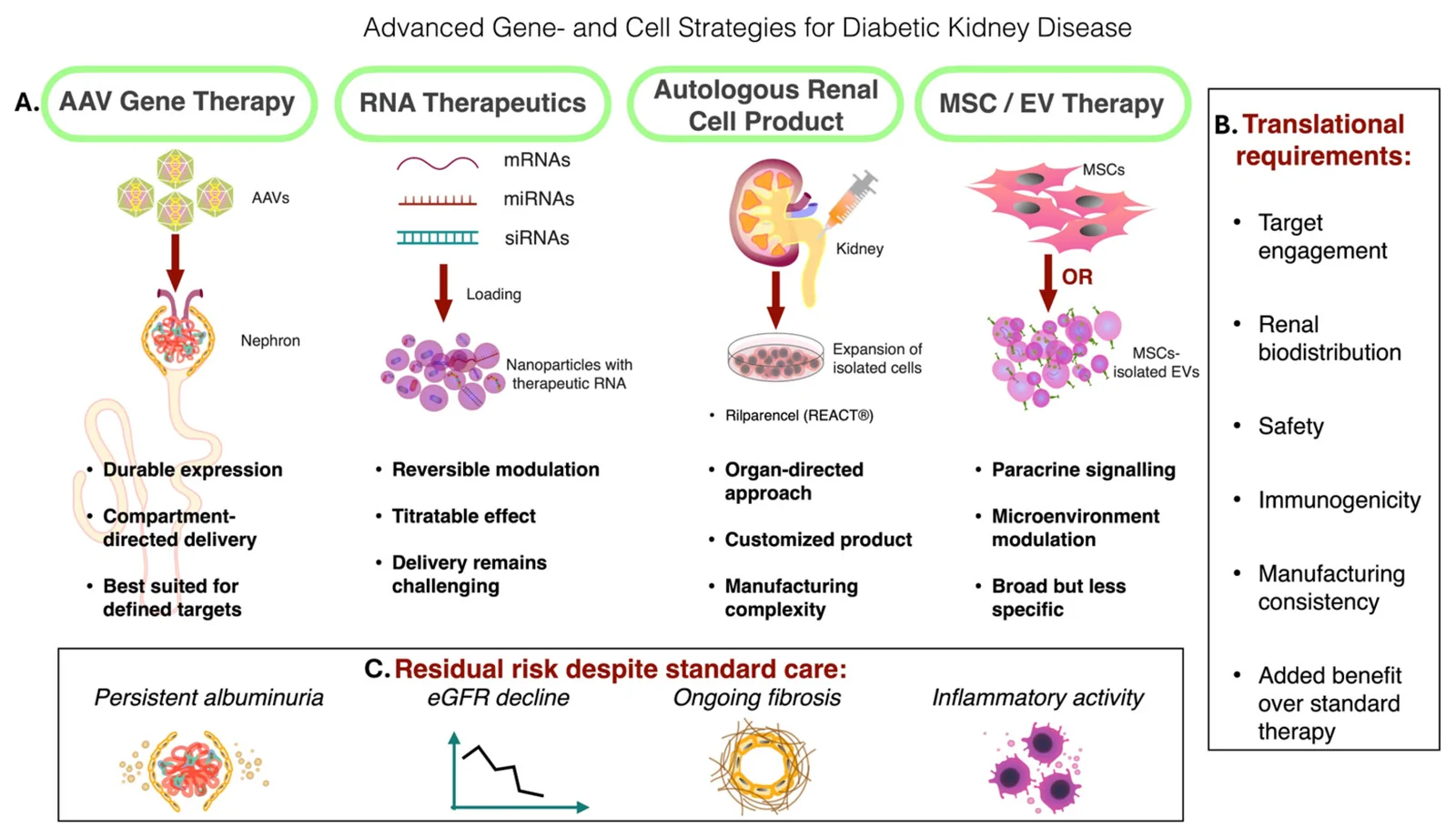
Advanced gene- and cell-based therapeutic strategies for diabetic kidney disease (DKD) and their translational considerations. (**A**) Schematic representation of modern therapeutic modalities directed at the nephron, including adeno-associated virus (AAV) gene therapy for durable expression, RNA therapeutics (mRNAs, miRNAs, siRNAs) utilizing nanoparticle-based delivery systems, autologous renal cell products (e.g., Rilparencel/REACT^®^), and mesenchymal stem cell (MSC) or MSC-derived extracellular vesicle (EV) therapies. (**B**) Critical translational requirements essential for clinical bench-to-bedside conversion, encompassing target engagement, renal biodistribution, biosafety, immunogenicity, manufacturing consistency, and therapeutic added benefits. (**C**) Clinical hallmarks of residual renal risk and progressive kidney injury despite standard-of-care treatment, characterized by persistent albuminuria, eGFR decline, ongoing tissue fibrosis, and sustained inflammatory activity.

Regulatory requirements depend on product composition, manipulation, persistence, and jurisdiction. Gene and cell products require integrated quality, nonclinical, and clinical evidence; EV products require case-specific classification and cannot be treated as exempt because they are cell-free [69,110]. Biodistribution, immunogenicity, dose justification, and risk-based long-term follow-up must be planned before pivotal testing [68,82]. No platform discussed here has demonstrated clinical efficacy sufficient for routine DKD use. Figure 2 summarizes the concepts; its proposed advantages should not be interpreted as validated clinical indications. Table 3 summarizes selected DKD studies, distinguishing actual enrollment and reported outcomes from planned recruitment and protocol-defined dosing, at the same time cell counts describe exposure, not equivalent potency across products.

## 6. Discussion: Translational Priorities for Advanced DKD Therapy

### 6.1. Lessons from Failed Translation

Past DKD trials illustrate different reasons for translational failure. In BEACON, bardoxolone methyl increased eGFR but did not improve the primary kidney/cardiovascular outcome and increased heart failure events, prompting early termination [112]. In the randomized anti-TGF-β1 antibody study, targeting a well-supported profibrotic pathway did not slow renal decline [113]. These are pharmacological examples, not trials of the platforms reviewed here, and their results cannot identify a single mechanism of failure. They show why an improved functional surrogate or strong mechanistic rationale does not guarantee net clinical benefit.

For advanced therapies, the analogous gaps are mismatched disease models, failure to reach the relevant human renal cells, insufficient or uncontrolled exposure, irreversible pathology at treatment initiation, and a product that changes during scale-up. Human tissue or organoids can reduce uncertainty about cell biology but cannot reproduce systemic immunity, perfusion, or long-term clinical safety. Negative and discordant findings should therefore guide product redesign and stopping decisions, rather than being dismissed as exceptions to otherwise promising animal results.

### 6.2. Patient Selection, Background Care, and Incremental Benefit

An initial efficacy-testing population could comprise adults with repeated evidence of eGFR decline and persistent risk despite stable, tolerated, indicated care. A run-in should document adherence, blood pressure, glycemia, medication changes, acute kidney injury, and alternative diagnoses. In type 2 diabetes, background treatment should include RAS blockade, an SGLT2 inhibitor, finerenone, and a GLP-1 receptor agonist as individually indicated and tolerated, with reasons for non-use recorded [5,6]. These drugs should not be withdrawn merely to increase the apparent effect of an experimental product. Randomization should balance major background drug classes, baseline eGFR, albuminuria, and diabetes type.

Timing should follow the intended mechanism. Barrier-protective strategies such as VEGFC require viable target cells; a tubular antifibrotic intervention requires accessible tissue with active, potentially modifiable injury. Advanced scarred disease may offer little substrate for repair, whereas low-risk early disease may not justify irreversible exposure. Non-albuminuric progression should not be excluded without mechanistic justification. These are proposed enrichment principles, not validated companion diagnostic criteria. Interactions with standard drugs, including overlapping hemodynamic, electrolyte, or immune effects, need prospective evaluation.

The expected incremental benefit cannot currently be quantified: no advanced platform has a definitive outcome trial on fully contemporary layered care. Relative effects from unrelated trials should not be added or multiplied. Early randomized studies should estimate dose–response and between-group effects with uncertainty; subsequent trials should be powered using realistic residual event rates and a prespecified clinically worthwhile benefit. Greater procedural burden, persistence, and cost require a correspondingly persuasive net benefit. Treatment burden and health-related quality of life should be measured alongside renal outcomes.

### 6.3. Endpoints, Biomarkers, and Trial Design

A phase 1/2 program should prioritize adverse events, feasible delivery, product exposure, and renal target engagement, with UACR and eGFR trajectories as supportive signals. A fall in albuminuria, an improved biopsy score, or a molecular signature alone does not prove prevention of kidney failure or improved survival. eGFR slope has empirical support as a surrogate under appropriate design conditions [113], but its interpretation depends on follow-up, acute hemodynamic changes, missing data, and the analytical estimand. A short within-patient pre/post slope comparison is particularly vulnerable to regression to the mean and changes in background care. Trials should prespecify total and chronic slope analyses when relevant, use mGFR or cystatin C to investigate creatinine-related discordance, and avoid equating a favorable exploratory slope with nephron regeneration.

For confirmatory assessment, randomized concurrent controls and sufficiently long follow-up are needed to evaluate sustained major eGFR decline, kidney failure requiring replacement therapy, and kidney death, using prespecified definitions. Cardiovascular events and all-cause mortality should be assessed separately rather than inferred from renal biomarker changes. Invasive products require a justified control strategy, blinded outcome assessment, and independent event adjudication; whether a sham procedure is ethical depends on its risk and the question being tested. Safety surveillance must extend beyond the period used to evaluate efficacy when exposure or biological effects persist [69,82].

Risk markers, predictive markers, and pharmacodynamic markers have different roles. UACR and eGFR describe risk and progression; neither proves target engagement. Candidate measures include bioactive Klotho with mineral metabolism monitoring, compartment-specific transgene expression where accessible, target transcript or protein modulation for RNA products, and product-linked functional or immune assays for cell/EV therapies. Urinary EV cargo and tissue-derived signatures are exploratory and may reflect shedding or cell loss rather than therapeutic delivery. None is currently a validated cross-platform predictor of response in DKD. Table 4 links proposed research priorities to measurable decision criteria.

### 6.4. Pediatric DKD and Special Populations

Adult DKD findings should not be extrapolated directly to children or adolescents. Pediatric disease differs in diabetes duration, pubertal physiology, growth, and the interpretation of albuminuria, while youth-onset type 2 diabetes may carry substantial early complication risk [114]. Advanced therapies lack an established pediatric DKD benefit–risk profile. Age-appropriate pharmacology, growth and developmental outcomes, consent/assent, and follow-up into adulthood are needed; durable gene expression creates particular uncertainty over a long remaining lifespan. Pregnancy requires reproductive and fetal risk assessment. Transplant recipients need separate consideration of alloimmunity and immunosuppression, and frail older adults or patients near kidney failure require explicit attention to competing mortality, treatment burden, and the time needed for benefit. These groups require dedicated evidence rather than automatic adoption of adult trial eligibility or efficacy estimates.

### 6.5. Limitations of This Review

This is a focused narrative synthesis with a targeted revision-stage verification, not an exhaustive systematic review. A prospective protocol and screening log for the original literature collection were not available for this revision; the update does not retrospectively establish such a protocol. Selection and publication bias remain possible; no independent duplicate screening, formal risk-of-bias scoring, or quantitative pooling was undertaken. Models, products, follow-up, and endpoints differ substantially, so the proposed priorities are a reasoned research framework rather than a comparative efficacy ranking. A future systematic review should address a narrower, prespecified question within a platform and population.

## 7. Conclusions

Advanced DKD therapies remain investigational. The strongest immediate contribution of the field is a set of testable mechanisms and delivery tools, together with preliminary clinical experience for selected cell products. Klotho and podocyte-directed VEGFC warrant product-specific preclinical validation; miR-21 requires resolution of compartment- and intervention-dependent effects before broad suppression is prioritized. MSC and renal cell programs require adequately controlled confirmation, while EV and renal mRNA products need stronger exposure, potency, and reproducibility evidence. No single target or platform can presently be designated clinically superior.

The practical sequence is to demonstrate activity in the intended human renal compartment, validate a reproducible product and dose, and then test incremental benefit in randomized add-on studies of patients with documented progression. Baseline UACR and eGFR establish clinical risk; candidate molecular markers require separate validation for selection or target engagement. Claims of kidney protection should ultimately be supported by durable functional preservation and appropriately designed kidney outcome studies, with safety and patient burden commensurate with the intervention. These requirements are more informative for prioritization than the magnitude of an isolated preclinical biomarker change.

## Figures and Tables

**Table 1:** Cross-platform comparison of feasibility, scalability, and proposed clinical niches.

Platform	Potential Advantage/Proposed Niche	Main Feasibility and Safety Constraints	Scalability/Clinical Maturity
AAV	Sustained protective expression if a defined renal compartment can be reached and persistent exposure is justified.	Poorly reversible exposure; immunity and restricted redosing; off-target distribution; route-related risk.	Specialized vector manufacture and lot comparability. DKD efficacy remains preclinical.
RNA/ASO/LNP	Time-limited pathway modulation or protein expression; possible dose adjustment for a modifiable injury program.	Productive cellular delivery and endosomal escape; off-target effects; cumulative carrier/immune toxicity.	Potential batch manufacture, with formulation and potency constraints. Renal DKD products remain experimental.
Allogeneic MSCs	Broad paracrine or immune modulation; banked product for selected inflammatory phenotypes, if confirmed.	Pulmonary sequestration; transient persistence; donor/lot variation; infusion and thrombogenic risk.	Expansion and cryobanking are feasible, but scale can change potency. Early human studies; confirmatory DKD efficacy remains unproven.
MSC-derived EVs	Acellular delivery of reparative cargo; potential repeat-dose intervention.	Uncertain active component, dose unit, renal delivery, and duration; soluble contaminants; repeat-dose safety.	Requires scalable isolation, purity, stability, and functional release assays. Predominantly preclinical.
Autologous renal cells	Organ-derived, patient-specific product with direct renal administration.	Biopsy/injection risk; variable source tissue; manufacturing delay and failure; uncertain durable mechanism.	Individualized production limits throughput. Clinical testing exceeds molecular platforms in maturity, but efficacy is unproven.

**Table 2:** Representative advanced-therapy evidence and the limits of inference. Proposed monitoring measures are distinguished from outcomes demonstrated in the cited studies.

Approach/Source	Evidence and Observed Outcomes	Limit of Inference	Proposed Monitoring/Next Evidence
AAV–TMD1 [72]	db/db mouse proof of concept; albuminuria, histology, and pathway changes.	No human DKD efficacy or established benefit on modern background care.	Target expression/activity; renal and systemic exposure; kidney function; vector safety.
Klotho [74,75,76]	Cell and rodent studies using different molecular forms and delivery methods.	Target support is broader than validation of any single gene-therapy product.	Active form and exposure; mineral metabolism; renal target engagement; functional outcomes.
Podocyte-directed VEGFC [77]	Experimental type 1 diabetes; improved barrier and glycocalyx readouts.	Early proof of concept; no demonstration of advanced fibrosis reversal.	Compartment specificity; local/systemic exposure; vascular safety; sustained function.
Kidney-directed AAV [60,78,79,80]	Transduction studies in animal and human-derived systems.	Delivery efficiency is not DKD efficacy; species and [104] route affect performance.	Productive expression versus vector presence; biodistribution; immunity; vector-only toxicity.
RNA/miR-21/CTGF [83,84,85,86,87,88,89]	Experimental target and injury responses; discordant miR-21 findings; negative anti-miR-21 trial in Alport syndrome.	No proven human DKD efficacy; non-DKD results cannot be transferred directly.	Target RNA/protein modulation; cell-specific uptake and endosomal escape; off-target, innate immune, and repeat-dose toxicity.
MSC/engineered MSC [25,96,97,98,99,104]	Human randomized studies and a single treated recipient; engineered products remain preclinical. See Table 3.	Heterogeneous products, related NEPHSTROM cohorts, attrition/reporting concerns, and no confirmed long-term benefit.	mGFR/eGFR; UACR; infusion/thrombotic events; potency; prespecified missing-data analysis.
MSC-derived EVs [108,109]	Predominantly preclinical; fibrosis and injury responses in diabetic mice.	Characterization standards do not demonstrate clinical efficacy or regulatory readiness.	Identity, purity, justified dose unit, cargo/activity attribution, biodistribution, repeat-dose safety.
Rilparencel [107]	Phase 2 dosing strategy trial; exploratory pre/post eGFR assessment.	No untreated in this study; procedure and manufacturing burdens.	Concurrent controlled efficacy; biopsy/injection safety; durable kidney outcomes.

**Table 3:** Selected clinical MSC studies in diabetic kidney disease: products, dosing, and limits of the clinical evidence.

Study/Population	Cell Product	Design and Sample Size	Dose per Infusion	Number, Route, and Follow-Up	Reported Findings/Evidence Limitations
Packham et al., 2016 NCT01843387 Type 2 DKD [96]	Allogeneic bone marrow mesenchymal precursor cells; rexlemestrocel-L	Randomized, placebo-controlled Actual *n* = 30: 10 per dose; 10 placebo	150 or 300 million cells	One IV infusion 60-week follow-up	Exploratory week 12 GFR trends; no treatment-related serious adverse events identified. Small safety study; no confirmed durable benefit.
NEPHSTROM, 2023 NCT02585622 Type 2 DKD [25]	Allogeneic CD362-selected bone marrow MSCs; ORBCEL-M	Randomized, placebo-controlled Actual *n* = 16: 12 active/4 placebo	80 million cells	One IV infusion 18-month follow-up	Slower eGFR decline; no significant mGFR benefit. No treatment-related serious adverse events identified. First dose cohort, not a separate trial.
NEPHSTROM, 2026 NCT02585622 Type 2 DKD [97,100]	ORBCEL-M; same product and trial as preceding row	Randomized, placebo-controlled Actual *n* = 13: 10 active/3 placebo	160 million cells	One IV infusion 18-month follow-up	No significant between-group eGFR or mGFR benefit. No infusion-related adverse events. Both cohorts were male-only; planned 240-million dose not tested.
Sabir et al., 2026 NCT07039318 Stage II–IV DKD [98,101]	Allogeneic bone marrow MSCs	Randomized, placebo-controlled Reported ITT *n* = 91; per protocol *n* = 78 (35/43)	30 million cells	One IV infusion 6-month follow-up	Per-protocol GFR signal; continuous ITT GFR comparison nonsignificant. Attrition, inconsistent arm counts, and retrospective registration limit interpretation.
Alatta et al., 2025 NCT03840343 Progressive DKD [99,111]	Autologous adipose-derived MSCs	Open-label phase 1 Actual *n* = 2 enrolled; only 1 treated	0.25 million cells/kg (administered)	Two infusions into one renal artery Baseline and month 4; month 3 planned	No related serious adverse events or procedural complications in the recipient. Uncontrolled pre/post slope observation; efficacy cannot be inferred.
NCT04125329 Diabetic nephropathy [102]	Allogeneic umbilical-cord MSCs	Early phase 1; single arm Planned *n* = 15	Approximately 1 million cells/kg	Three IV infusions Weeks 0, 4, 8 Assessment to week 60	Protocol only; status unknown and no registry results posted. Last posted update: 21 April 2021.
NCT04216849 Type 2 diabetic nephropathy [103]	Allogeneic umbilical cord MSCs	Phase 1/2; randomized, placebo-controlled Planned *n* = 54	1.5 million cells/kg	Five IV infusions Weeks 0, 8, 16, 24, 32 Assessment to week 48	Protocol only; status unknown and no registry results posted. Last posted update: 17 December 2020.

**Table 4:** Proposed research priorities and decision criteria for advanced DKD therapy.

Research Priority	Candidate Population/Biological Question	Decision Evidence Required
Klotho/barrier-protective VEGFC	Progressive disease with viable target cells; can sufficient active product reach the intended compartment?	Defined active form and exposure; human cell activity; systemic/mineral or vascular safety; reproducible preclinical functional benefit before efficacy trials.
RNA-mediated fibrosis/inflammation modulation	Active, accessible injury program; is partial, time-limited modulation beneficial in the relevant cell type?	Productive delivery; target RNA/protein response; resolve contradictory miR-21 effects; repeat-dose safety; randomized functional outcomes.
MSC/EV products	Selected inflammatory or endothelial phenotype; does the released product have reproducible relevant activity?	Mechanism-linked potency; lot comparability; exposure/activity attribution; concurrent controls; no inference of cell replacement from biomarker changes.
Autologous renal-cell products	Progressive adult DKD with an acceptable biopsy/injection risk and sufficient time to benefit.	Reproducible manufacture; appropriate randomized control; procedure safety; sustained functional benefit and kidney outcomes.
Shared trial framework	Repeated progression despite stable, indicated, tolerated care; stratification by diabetes type, eGFR, UACR, and background therapy.	Separate risk, predictive, and pharmacodynamic markers; blinded outcomes; prespecified analyses; assess kidney failure, mortality, safety, and patient burden.

## Data Availability

No new data were created or analyzed in this study. Data sharing is not applicable to this article.

## Abbreviations

The following abbreviations are used in this manuscript:

AAV	adeno-associated virus
ADA	American Diabetes Association
AGE	advanced glycation end product
ASO	antisense oligonucleotide
CCN2	cellular communication network factor 2
CKD	chronic kidney disease
CTGF	connective tissue growth factor
DKD	diabetic kidney disease
DN	diabetic nephropathy
eGFR	estimated glomerular filtration rate
ER	endoplasmic reticulum
EV	extracellular vesicle
GFR	glomerular filtration rate
GLP-1	glucagon-like peptide-1
HK-2	human kidney-2 cell line
ITT	intention-to-treat
IV	intravenous
KDIGO	Kidney Disease: Improving Global Outcomes
LNP	lipid nanoparticle
mGFR	measured glomerular filtration rate
miRNA/miR	microRNA
MISEV2023	Minimal Information for Studies of Extracellular Vesicles 2023
mRNA	messenger RNA
MSC	mesenchymal stromal cell
NEPHSTROM	Novel Stromal Cell Therapy for Diabetic Kidney Disease
NF-κB	nuclear factor-κB
NLRP3	NLR family pyrin domain-containing 3
NOD	non-obese diabetic
NRF2	nuclear factor erythroid 2-related factor 2
RAS	renin–angiotensin system
REACT	renal autologous cell therapy
RNA	ribonucleic acid
ROS	reactive oxygen species
SGLT2	sodium–glucose cotransporter 2
siRNA	small interfering RNA
STZ	streptozotocin
TGF-β	transforming growth factor-β
TIMP3	tissue inhibitor of metalloproteinases 3
TMD1	thrombomodulin domain 1
UACR	urinary albumin-to-creatinine ratio
VEGFC	vascular endothelial growth factor C
